# NLRP3 inflammasome as a potential treatment in ischemic stroke concomitant with diabetes

**DOI:** 10.1186/s12974-019-1498-0

**Published:** 2019-06-07

**Authors:** Pu Hong, Ruo-Nan Gu, Feng-Xian Li, Xiao-Xing Xiong, Wen-Bin Liang, Zhi-Jian You, Hong-Fei Zhang

**Affiliations:** 1 Department of Anesthesiology, Zhujiang Hospital of Southern Medical University, Guangzhou, Guangdong People’s Republic of China; 2 Department of Neurosurgery, Renmin Hospital of Wuhan University, Wuhan, Hubei People’s Republic of China; 3 Cardiac Electrophysiology Lab, University of Ottawa Heart Institute, Ottawa, Ontario K1Y 4 W7 Canada; 4 Department of Cellular and Molecular Medicine, University of Ottawa, Ottawa, Ontario K1Y 4 W7 Canada; 5 Department of Anesthesiology, Shenzhen SAMII Medical Center, Shenzhen, Guangdong People’s Republic of China

**Keywords:** Inflammation, Apoptosis, Interleukin-1β, Diabetes mellitus, Stroke

## Abstract

The NLRP3 (nucleotide-binding oligomerization domain-like receptor [NLR] family pyrin domain-containing 3) inflammasome is a member of the NLR family of innate immune cell sensors. These are crucial regulators of cytokine secretions, which promote ischemic cell death and insulin resistance. This review summarizes recent progress regarding the NLRP3 inflammasome as a potential treatment for ischemic stroke in patients with diabetes, two complicated diseases that often occur together. Stroke worsens glucose metabolism abnormalities, and the outcomes after stroke are more serious for diabetic patients compared with those without diabetes. Inflammation contributes to organ injury after ischemic stroke and diabetes. Recent research has focused on inhibiting the activation of inflammasomes and thus reducing the maturation of proinflammatory cytokines such as interleukin (IL)-1β and IL-18. Studies suggest that inhibition of NLRP3 prevents or alleviates both ischemic stroke and diabetes. Targeting against the assembly and activity of the NLRP3 inflammasome is a potential and novel therapy for inflammasome-associated diseases, including ischemic stroke concomitant with diabetes.

## Introduction

Ischemic stroke (also called cerebral ischemia) is a leading cause of death and disability worldwide, currently with about 30 million sufferers. In 2015, deaths due to stroke accounted for 11.8% of total deaths, making it the second leading global cause of mortality, behind heart disease [1]. From 2000 to 2008, the rate of incidence in low-to-middle-income countries exceeded that of high-income countries [2].

Stroke may be ischemic (~ 80%) or hemorrhagic (~ 20%) and the middle cerebral arteries are the most common site when ischemia occurs [3, 4]. Diabetes has also increasingly become a major public health problem, affecting an estimated 642 million people by 2040 [5]. Thus, ischemic stroke and diabetes mellitus represent a severe socioeconomic burden.

Strong evidence indicates that diabetes and ischemic stroke are related bidirectionally. Ischemic stroke can cause disorders of glucose metabolism, which in turn delays the recovery of brain function after stroke [6]. Hyperglycemia is found in approximately 40% of patients with acute ischemic stroke [7]. According to 33 studies altogether, of patients with ischemic stroke and hyperglycemia upon admission, 39–83% were diabetic and 8–63% were non-diabetic [8]. Of patients admitted to hospital for first-ever ischemic stroke, 36.3% were diabetic [9]. The risk of ischemic stroke in patients with diabetes is almost 2-fold that of patients without diabetes, and after ischemic stroke, diabetic patients suffer a larger infarct size, more obvious cerebral edema, poorer clinical outcomes, and a higher risk of mortality [10]. Studies using rats or mice models of these diseases also support this conclusion [11, 12].

Brain damage in ischemic stroke and diabetes is aggravated by an excessive inflammatory cascade reaction, and continuous inflammation is the primary cause of tissue damage and organ dysfunction. Increasing evidence suggests that inflammation promotes the progression of diabetes and ischemic stroke [13, 14]. The cytokine interleukin-1β (IL-1β) has very strong pro-inflammatory effects on a variety of cell types and is implicated in the pathogenesis of numerous inflammatory diseases, including stroke, diabetes, and genetic auto-inflammatory disorder. Given the prominent role of IL-1β in inflammation, some studies have focused on the activation and regulation of IL-1β-driven pro-inflammatory cascades by inflammasomes [15, 16].

The nucleotide-binding oligomerization domain-like receptor (or, NOD-like receptor, NLR) family comprises innate immune cell sensors that are involved in the secretion of cytokines. The NLRP3 (NLR family pyrin domain-containing 3) inflammasome is a subtype of the NLR family encoded by the gene NLRP3 [17]. The NLRP3 inflammasome, also known as cryopyrin or NALP3, has an essential role in the damage caused by inflammation associated with ischemic stroke and type 2 diabetes mellitus (T2DM) [18, 19]. Previous studies have provided much evidence that downregulation of NLRP3 may help treat both diabetic patients and ischemic stroke patients [20, 21]. However, there are many patients that are afflicted by both T2DM and ischemic stroke, and the possible effects of NLRP3 regulation for these patients are not well understood.

Given the apparent synergistic adverse effects of ischemic stroke and diabetes, it is important to determine how these diseases are related and explore potential therapies such as the NLRP3 inflammasome. In this review, we summarize the present understanding of the composition, activation, and regulation of the NLRP3 inflammasome, and its potential therapeutic roles in ischemic stroke occurring concomitant with diabetes (referred to as diabetic-stroke).

### Composition of NLRP3 inflammasome

The first line of host defense against disease is the innate immune system, which relies on receptors that sense the molecular patterns associated with microbes and endogenous or exogenous pathogens and the damage they cause [15]. The NLRs are characterized by a central nucleotide binding and oligomerization domain termed NACHT (reflecting its major proteins). NACHT is an acronym for NAIP (neuronal apoptosis inhibitor protein), C2TA (class 2 transcription activator, of the MHC), heterokaryon incompatibility, and TP1 (telomerase-associated protein 1) [17]. The central NACHT is flanked by a C-terminal leucine-rich repeat (LRR) and N-terminal caspase recruitment or pyrin domains (CARD and PYD, respectively) [22, 23].

NLRs, including NLRP1, NLRP3, and others, are involved in the assembly of a multiprotein platform that has been termed the inflammasome [24, 25]. The inflammasome contains the sensor molecule NLR, as well as pro-inflammatory caspase (pro-caspase 1, pro-caspase 5, or both) and adaptor proteins. Inflammasomes act as a roving security force inside the cell, detecting a variety of danger signals such as bacterial RNA or bits of bacterial flagellin [26].

**Fig. 1:**
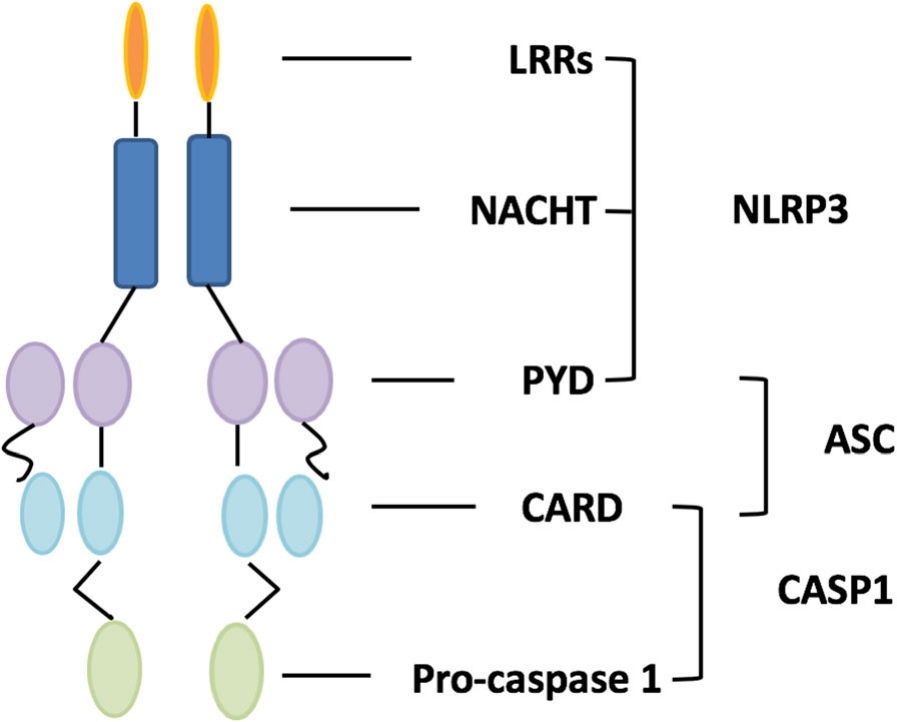
Schematic of the NLRP3 inflammasome. ASC apoptosis-associated speck-like protein, CARD caspase activation and recruitment domain (light blue oval), LRR leucine-rich repeat (orange elongated ovals), NACHT nucleotide-binding and oligomerization domain (blue rectangles), PYD pyrin domain (purple oval)

The NLRP3 inflammasome in particular has been well characterized. The NLRP3 inflammasome is a multiprotein complex in cells, with the following core proteins: three domains of NLRP3 (NACHT, LRR, and PYD domain-containing protein 3); the adaptor protein apoptosis-associated speck-like protein containing a CARD (ASC); and inflammatory caspase 1 (cysteine-dependent aspartate-directed protease 1) (Fig. 1). The major function of NLRP3 inflammasomes is to recognize a wide variety of danger signals that are due to exogenous infection and internal damage. These include diverse ligands and stimuli such as uric acid and even hexokinase. Through recognition of damage-associated molecular patterns, the NLRP3 inflammasome, with its ASC and pro-caspase 1 components, promotes the activation of caspase 1 and the processing of cytoplasmic targets, including IL-1β and IL-18 [27, 28].

Elevated levels of IL-1β may be a contributing factor to insulin insensitivity in obese patients and brain dysfunction observed in the diabetic after stroke. Because activation of the metabolic signaling pathway, especially IL-1R signaling, is closely associated with insulin receptor substrate 1 (IRS-1), it enhances the expression of inflammatory mediators, inducing endoplasmic reticulum stress and oxidative stress. It also directly triggers insulin resistance by promoting expression of tumor necrosis-factor-α (TNF-α), known as an inducer of insulin resistance [29]. IL-1β also is the hallmark of macrophage/microglia and other immune cell activation in the diabetic rat brain, indicating exacerbation of inflammatory responses in ischemic injury [12, 30].

### Regulation of the NLRP3 inflammasome

Chronic inflammatory responses by the NLRP3 inflammasome are involved in the course of various diseases such as gout, atherosclerosis, Alzheimer’s disease, T2DM, and stroke. In general, activation of the NLRP3 inflammasome requires 2 signaling pathways, priming and activating. The initial priming signal results in the transcription of pro-IL-1β and pro-IL-18, while the activating signal triggers the formation of subsequent NLRP3 inflammasomes.

The initial priming signal induces the activation of nuclear factor (NF)-κB, which can be mediated through pattern-recognition receptors, cytokine receptors [31, 32], or factors such as high extracellular glucose [33], extracellular ATP [34], *Staphylococcus aureus* [35], cholesterol [36], and others (Table 1). Nevertheless, Yu et al. [49] reported that a two-step model of activation of the NLRP3 inflammasome is not applicable under some circumstances. They showed that mitochondria are a key component downstream of NLRP3 activation, and accompanied by complex cellular changes, NLRP3 inflammasome activation may be related to mitochondrial dysfunction [49].

**Table 1:** Literature regarding the regulation of the NLRP3 inflammasome

		References
Activation	High extracellular glucose	[33]
	Hexokinase	[28, 37]
	Extracellular ATP (eATP)	[34, 38]
	β-amyloid	[39]
	*S. aureus*	[35]
	Cholesterol	[36]
	Uric acid crystal	[40]
	Alum	[41]
	Low K^+^	[42]
Negative regulation	Autophagy	[43]
	Nitric oxide	[44]
	Type I IFNs	[45]
	Measles virus (MV)	[46]
	MiR-233	[47]
	Effector and memory T cells	[48]

**Fig. 2:**
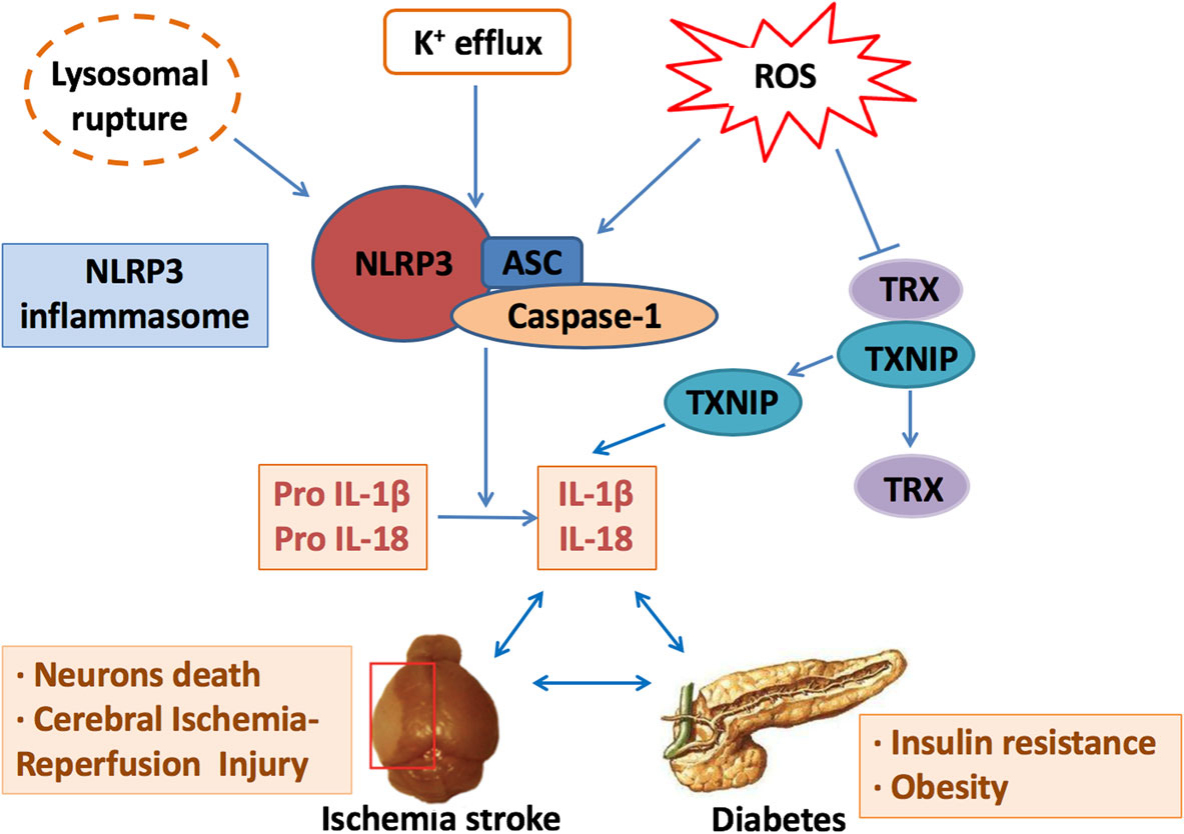
Schematic overview of NLRP3 inflammasome activation mechanisms in ischemic stroke concomitant with diabetes. NLRP3 inflammasome has a crucial role in diabetes and ischemic stroke based on three canonical hypotheses—reactive oxygen species (ROS), lysosomal rupture, and cellular potassium efflux. These mechanisms may collectively activate caspase 1, which mediates the release of cytokines such as IL-1β and IL-18. Increased ROS are sensed by a complex of thioredoxin (TRX) and TRX-interacting protein (TXNIP) that can induce the dissociation of the complex. TXNIP binds to the LRR region of NLRP3, leading to NLRP3 inflammasome activation and the secretion of mature IL-1β and IL-18. The NLRP3 inflammasome is a platform for IL-1β and IL-18 production. After activation of the NLRP3 inflammasome, cells secrete a great many proinflammatory cytokines, which aggravates insulin resistance (in diabetes) and neuronal death (ischemic stroke)

Indeed, while many activators of the NLRP3 inflammasome have been identified, but the mechanism of activation has not been fully elucidated. The 3 classical hypotheses involve reactive oxygen species (ROS), lysosomal rupture, and cellular potassium efflux [25, 36, 50–52] (Fig. 2). A recent finding now suggests that oxidative stress is not only a crucial driver of inflammation, but these disturbances also induce activation of the NLRP3 inflammasome in diabetes and complications of diabetes [53].

Although infection, tissue damage, and metabolic dysregulation can trigger activation of the NLRP3 inflammasome, the host can control against damage caused by the resulting inflammation via a mechanism of negative regulation [51, 54]. The degradative process of autophagy regulates innate immune responses and NLRP3-dependent inflammation by conserving mitochondrial integrity [43]. In general, autophagy is beneficial for cells, but under conditions of prolonged stress, autophagy can lead to cell death that is distinct from apoptosis. Autophagy may control inflammation through the degradation of pro-IL-1β, thus regulating IL-1β levels [55]. In addition, autophagy can restrain inflammasome activity by decreasing the generation of ROS [56].

Also of note, nitric oxide acting as an immunomodulatory molecule can inhibit activation of the NLRP3 inflammasome via stabilization of mitochondria, in both mice and humans [44]. Type I interferons diminish levels of intracellular pro-IL-1β by inducing production of the anti-inflammatory cytokine IL-10, dependent on the transcription factor signal transducer and activator of transcription 1 (STAT1) [45]. Other negative regulators of the NLRP3 inflammasome also exist, such as certain viruses, microRNA, and T cells [46–48].

## Potential for targeting the NLRP3 inflammasome for treating ischemic stroke concomitant with diabetes

### Ischemic stroke and T2DM

Diabetes is often accompanied by risk factors that contribute to stroke and other cardiovascular diseases [57, 58]. Although the pathophysiology remains vague, patients with diabetes appear to have a higher susceptibility to ischemic stroke and with poorer outcomes. About 14.18% of patients with ischemic stroke also have diabetes. In Chinese patients with diabetes, the percentage of deaths due to ischemic stroke is higher than that from ischemic heart disease, whereas in western countries the opposite is true [59, 60]. Diabetes is an independent risk factor of stroke, with a risk that is ~ 2-fold that of non-diabetic individuals (relative risk 2.2, 95% CI 1.9–2.6) and with associated poorer long-term post-stroke complications such as large vessel infarction, cognitive impairment, and even dementia [6, 10, 61]. Hyperglycemic rats were also found to have significantly larger infarcts: a systematic review reported that in rat models of middle cerebral artery occlusion (MCAO), hyperglycemic rats had infarcts that were 94% larger [11].

Treatment of T2DM can reduce stroke risk, but a systematic meta-analysis by Liu et al. [62] concluded that sulfonylurea treatment may contribute a significant risk of stroke in patients with T2DM. Therefore, drugs that treat ischemic stroke comorbid with diabetes are required and would have clinical implications for both treatment and management. Because of the detrimental role of sustaining sterile inflammation in diabetes and ischemic stroke, efforts have been made to develop NLRP3 inflammasome inhibitors against these diseases, including MCC950, glyburide, and others (Table 2). These anti-inflammatory biological agents have shown promise in animal models with either diabetes or ischemic stroke, or humans with these diseases. Specifically, in T2DM, these agents for treating inflammasome-mediated disease have been associated with improvements in glucose tolerance and insulin sensitivity and in slowing progression. In ischemic stroke, they putatively reduce neurological deficits, infarct volume, and brain edema and improve long-term outcomes.

**Table 2:** Potential therapy targets of the NLRP3 inflammasome in stroke and type 2 diabetes

	Animal model/patient	Proposed mechanism	Outcomes	References
MCC950	In mice in vivo and human cells ex vivo; pig model of myocardial infarction	Selective inhibition of NLRP3 inflammasome activation; dose-dependently inhibited IL-1β	The amount of CASP1 (an auto-processed fragment of CASP1) is dose-dependently reduced in supernatants from MCC950-treated BMDM and PBMC; infarct size as a percentage of the area at risk is significantly lower in both treatment groups compared with the control group.	[63, 64]
Glyburide	Patients with FCAS; P2X7^¯/¯^ mice	Inhibit ATP-sensitive K^+^ channels; downstream of P2X7	Glyburide blocks the rapid, CASP1-dependent cell death that occurs when BMDMs are treated with LPS and ATP.	[65]
IVIG	Mouse model of focal ischemic stroke	Downregulation of the pro-inflammatory cytokines IL-1β and IL-18; upregulation of Bcl-2	Administration of IVIG to mice subjected to experimental stroke significantly reduces brain infarct size and eliminates mortality; IVIG significantly decreases GD-induced neuronal cell death.	[66, 67]
Anakinra	Diabetic patients	IL-1 receptor antagonist	Proinsulin-to-insulin ratio was lower in anakinra-treated patients cf. placebo-treated patients.	[68, 69]
Parthenolide and Bay 11-7082	NLRP3^¯/¯^ macrophages	Inhibits ATPase activity of NLRP3	Blocking macrophage cell death in a dose-dependent manner.	[70]
MNS	WT, Syk^¯/¯^ mice	Inhibits NLRP3 ATPase activity	MNS inhibits the production of mature IL-1β in the cell supernatant as shown by immunoblotting.	[71]
Omega-3 fatty acids	HFD-treated mice, NLRP3^¯/¯^mice	Blocking metabolic stress-induced NLRP3 inflammasome activation	Reduces fasted glucose concentrations and improves glucose tolerance and insulin sensitivity.	[72]
NaB	Diabetic db/db mice	Inhibits NLRP3 inflammasome pathway	Improves glucose control and decreases the protein levels of NLRP3 & IL-1β.	[73]
γT3	Diabetic db/db mice	Blocking of NLRP3 inflammasome priming and activation	γT3 preserves insulin sensitivity and ameliorates the progression of type 2 diabetes.	[74]
ILG	H-treated mice	Inhibits NLRP3 inflammasome activation	ILG attenuates HFD-induced obesity, hypercholesterolemia, and insulin resistance.	[75]
RSV	T2DM rat model	Inhibits the activation of NLRP3 inflammasome via TXNIP	Alleviates DM-induced left-ventricular dysfunction and myocardial remodeling by inhibiting NLRP3.	[76]
A151	Rat model of SHR-SP	Reduces the maturation of IL-1β and CASP1 and exp of NLRP3 and iNOS in response to LPS and OGD stimulation	A151 reduces ischemic brain damage and NLRP3 mRNA levels in SHR-SP rats that have undergone pMCAO.	[77]
Chrysophanol	tMACO mouse model	Suppresses exp of NLRP3, CASP1, and IL-β	Reduces neurological deficits, infarct volume, and brain edema and ameliorates BBB permeability.	[78]
GSPB2	Diabetic db/db mice	Suppresses the upregulation of NLRP3	Notably attenuates levels of IL-1β and NLRP3 increased in a diabetic pancreas.	[79]
UMB	MCAO rat model	Reduces exp of TXNIP	UMB reduces the infarct volume and attenuated the production of IL-β and IL-18 by suppressing the exp of NLRP3 inflammasome.	[80]
Sinomenine	MCAO/R mouse model	Inhibits AMPK-mediated NLRP3 inflammasome activation	SINO reduces neuronal loss and attenuates the release of inflammatory cytokines after MCAO.	[81]
NADPH+ apocynin	MCAO/R mouse model	Inhibits activation of pro-inflammatory transcription factors NF-κB and its down-stream NLRP3 inflammasome pathway	NADPH and apocynin significantly reduce infarct volume, improve post-stroke survival, and recovery of neurological functions in MCAO/R mouse model.	[82]

*BMDM* bone marrow-derived macrophage, *CASP1* caspase 1, *exp* expression, *FCAS* familial cold autoinflammatory syndrome, *γT3* gamma-tocotrienol, *GSPB2* grape seed procyanidin B2, *HFD* high-fat diet, *ILG* isoliquiritigenin, *IVIG* intravenous immunoglobulin, *MCAO* middle cerebral artery occlusion, *MNS* 3,4-methylenedioxy-β-nitrostyrene, *NaB* sodium butyrate, *PBMC* peripheral blood mononuclear cell, *RSV* rosuvastatin, *SHR-SP* stroke-prone spontaneously hypertensive, *UMB* umbelliferone, *WT* wild type

### NLRP3 inflammasome in ischemic stroke

The treatment of ischemic stroke relies on the restoration of blood flow in the ischemic area. Yet, in a portion of ischemic brain tissues, renewed perfusion can exacerbate damage or dysfunction, leading to cerebral ischemia-reperfusion injury (I/R). Excessive inflammation has a prominent role in aggravating I/R and slowing or preventing recovery of brain function. Some studies have shown that I/R significantly increased levels of NLRP1, NLRP3 inflammasome proteins, IL-1β, and IL-18 in the ipsilateral brain tissues of I/R-model mice or stroke patients [66, 83]. The inflammasome is now seriously considered an essential component in the pathological progression of ischemic stroke and I/R [84, 85].

The risk of stroke is influenced by the presence of diabetes mellitus, hypertension, smoking, physical activity, diet, psychosocial factors, abdominal obesity, alcohol, cardiac causes, and apolipoproteins [86]. In addition, ischemic stroke, diabetes, obesity, and others are known to promote inflammation in the blood vessel wall. Associated with endothelial cell inflammation, these factors can increase levels of TNF-α, thus causing cerebrovascular endothelial damage [87]. Ischemia is also associated with elevated levels of inflammasome proteins, IL-1β, and IL-18. Gustin et al. [88] indicated that, in mouse brain, the NLRP3 inflammasome and secretion of IL-1β is limited to the microglial compartment, but not astrocytes.

The expressions of several inflammatory genes, such as pro-inflammatory cytokines IL-1β and chemokines, which are highly toxic to neurons, are significantly higher in the diabetic mouse brain after transient MCAO. Uncontrolled inflammation is thought to be a contributing mediator to exacerbate post-stroke damage in the diabetic mouse brain [12, 89]. Under neuroinflammatory conditions, inflammasome activation is by way of the microglia in the brain. Since the brain consumes a great deal of glucose and oxygen, in the early period of a stroke rapid disturbances in the blood supply lead to the development of an ischemic infarct, with accompanying neuronal necrosis and the generation of damage-associated molecular patterns. This in turn leads to the NLRP3-mediated inflammatory response, affecting the host’s immune balance and exacerbating the effects of ischemic stroke [19, 21].

During ischemic inflammation and the innate immune response, inflammasome-signaling pathways may act as key mediators. As reported by Fann et al. [90], NF-κB and mitogen-activated protein kinase (MAPK) signaling pathways are important to the expression and activation of inflammasomes, including NLRP1 and NLRP3, in primary cortical neurons under ischemic conditions. These authors were the first to show that activation of either the NF-κB or MAPK signaling pathway is associated with elevations of these inflammasome-related proteins in ischemic neurons. During I/R, the generation of ROS can stimulate brain inflammation and NLRP3 inflammasome activation, inducing more brain cell damage, brain edema, and brain dysfunction [91, 92]. A mini-review of Tong et al. [84] discussed an association between the regulatory mechanisms of the NLRP3 inflammasome and the development of stroke. The cyclic reaction mechanism that activates NLRP3 also aggravates atherosclerosis, leading to stroke. Abulafia et al. [93] demonstrated the formation of the inflammasome complex and activation of downstream inflammatory responses in mice under ischemic stroke conditions.

The NLRP3 inflammasome may mediate neuronal and glial cell death in ischemic stroke through a number of mechanisms, by increasing the production and secretion of the pro-inflammatory cytokines IL-1β and IL-18 and through the pleiotropic effects of cleaved caspase 1 in mediating brain cell apoptosis [94]. Most importantly, increasing evidence in mouse models indicates that inhibition of the NLRP3 inflammasome may protect against neurological deterioration after ischemic stroke and decrease the infarct volume [19, 95].

As mentioned above, the NLRP3 inflammasome is an important factor in inflammatory injury after stroke, but whether it is the leading factor for deteriorating diabetic-stroke remains an unanswered question. Based on this background, we previously established a mouse model of T2DM and MCAO and found that treatment with the NLRP3-specific inhibitor MCC950 alleviated neurological deficits and improved long-term survival [96]. In addition, mRNA levels of IL-1β/NLRP3 were significantly elevated in the ischemic brain, but were lower when treated with MCC950. Similar to our findings, a study by Liu et al. [97] indicated that MCC950 offers the benefit of reducing the disruption of the blood-brain barrier and cell death after intracerebral hemorrhage.

**Fig. 3:**
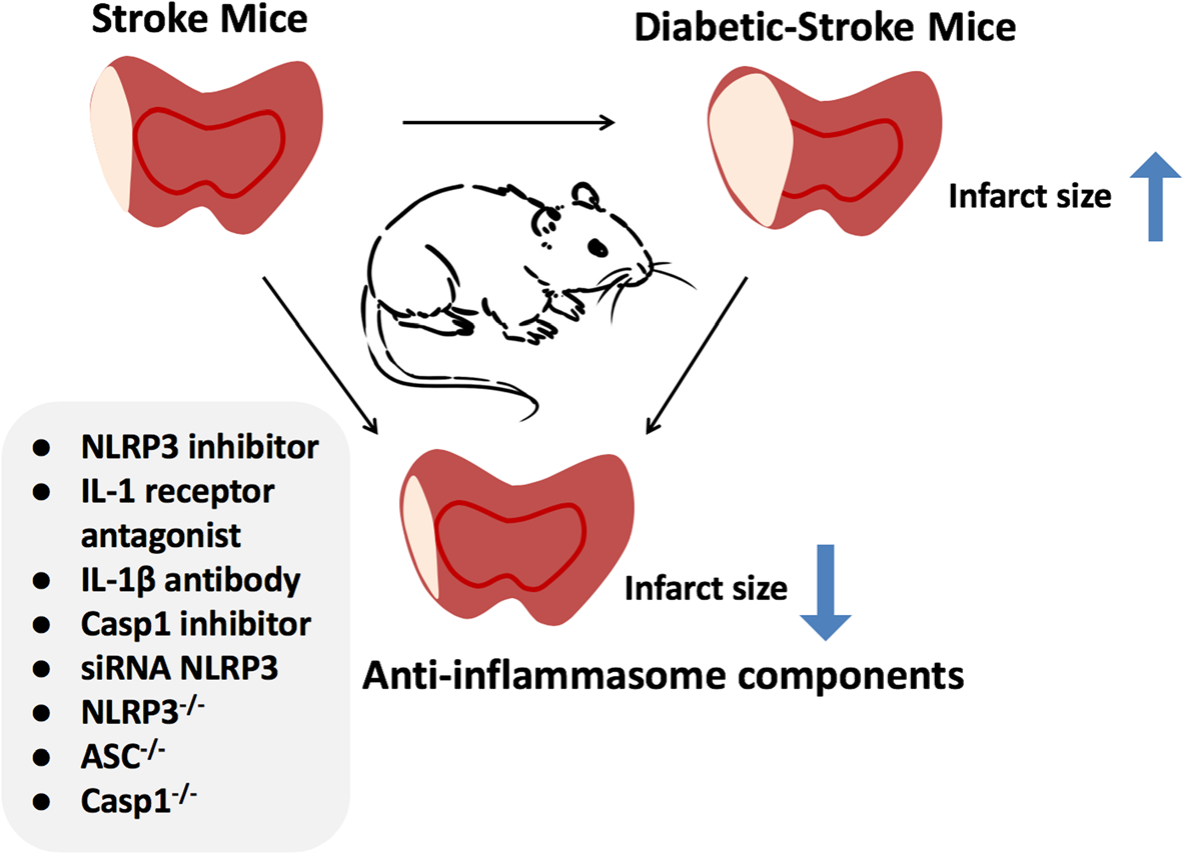
Novel treatment strategies to reduce infarct size by inhibiting the NLRP3 inflammasome in a mouse model of middle cerebral artery occlusion. The cerebral infarction of mice with stroke or diabetic-stroke, and treated or genetically modified, is shown. Diabetic mice had significantly larger infarcts. The activation of NLRP3 inflammation is a crucial step after stroke and diabetes. Targeting NLRP3 inflammasome formation or its products (IL-1β) by using NLRP3-specific siRNA to genetically delete any inflammatory component (NLRP3, ASC or caspase 1), or a selective NLRP3 inhibitor, is an important new avenue in stroke treatment

Besides MCC950, sinomenine is an anti-inflammatory molecule that provides neuroprotection by inhibiting the NLRP3 inflammasome via adenosine 5′-monophosphate (AMP)-activated protein kinase (AMPK) signaling in the mouse brain after MCAO [81]. With the high aggregation of IL-1β in the ischemic site of the brain, a receptor antagonist (IL-1RA) can block activation of IL-1β, partly due to activation from the NLRP3 inflammasome. In a randomized controlled phase 2 trial for ischemic stroke, IL-1RA was found safe and provided some benefit for patients presenting within 5 h of the onset of ischemic stroke [98]. In mouse experiments, diabetic stroke can be treated with the selective NLRP3 inhibitor MCC950. Targeting NLRP3 inflammasome formation or its products (IL-1β) using NLRP3-specific small interfering RNA (siRNA) to delete genetically any inflammatory component (NLRP3, ASC, or caspase 1) is also a possible strategy to reduce infarct size (Fig. 3).

NLRP3 inflammasome blockers have been successfully used in clinics as anti-inflammatory drugs (Table 3). These include the following: IL-1β receptor antagonist (anakinra), IL-1β antibody (canakinumab), caspase 1 inhibition drugs (ritonavir), P2X7 receptor antagonists (AZ11645373), NLRP3 inhibitor drugs (atorvastatin), and K_ATP_ channel blocker (glibenclamide). Unfortunately, there is a lack of clinical studies regarding NLRP3 inflammasome-specific treatment in patients with diabetes complicated with stroke. Therefore, we only list a few clinical studies that can be referred to for the treatment of diabetes or stroke. A randomized, controlled, double-blind clinical study of diabetic patients with stroke that focuses on inhibiting the NLRP3 inflammasome is anticipated.

**Table 3:** Clinical trial of targeting directly/indirectly the NLRP3 inflammasome in stroke and diabetes

	Target	Drug regimen	Main finding	Reference
Stroke patients	IL-1 receptor antagonist (IL-1Ra) (anakinra)	100 mg twice daily for 3 day in patients presenting within 5 hours of the ischemic stroke onset	Reduction of plasma IL-6 and plasma CRP for the first 3 days	[98]
	IL-1beta antibody (canakinumab)	A dose of 150 mg every 3 months	Lower rate of recurrent cardiovascular events	[99, 100]
	NLRP3 inhibitor drugs (atorvastatin)	80 mg/day	Lower plasma levels of IL-1β, CRP, TNF-a, and other immune-inflammatory markers at 72 h and 7 days after stroke	[101]
Diabetic patients	IL-1 receptor antagonist (IL-1Ra) (anakinra)	Lasting a 52-week treatment	Improvement of the fasting ratio of proinsulin to insulin (PI/I); reduction of plasma IL-6 and CRP	[69]
	IL-1beta antibody (canakinumab)	Canakinumab 150 mg	Improving ISR relative to glucose 0–0.5 h in patients treated with insulin	[102]
	IL-1beta antibody (LY2189102)	LY2189102 (0.6, 18, and 180 mg) administered weekly for 12 weeks	Reduction of hemoglobin A1c (HbA1c), fasting and postprandial glucose, hs-CRP, and IL-6	[103]

*IL-1Ra* IL-1 receptor antagonist, *CRP* C-reactive protein, *hs-CRP* high-sensitivity C-reactive protein, *HbA1c* hemoglobin A1c

Currently, NLRP3 drug development for the treatment of diabetes and stroke is still in the initial stage and no selective NLRP3 blockers are available in a clinical setting. Studies with a selective NLRP3 inhibitor to prevent or cure stroke occurring concomitant with diabetes are eagerly awaited. Although there have been only animal (mouse) experiments to verify the role of NLRP3 in the diabetic-stroke brain, NLRP3 inflammasome inhibitors may likely mitigate the disease outcome of patients with ischemic stroke concomitant with diabetes.

### NLRP3 inflammasome in T2DM

Diabetes is a metabolic disease. Moreover, T2DM is clearly related to obesity and insulin resistance. Chronic inflammatory responses can enhance the risk of insulin resistance in T2DM. An association between inflammasomes and T2DM is increasingly accepted. The NLRP3 inflammasome is activated by a variety of pathways, which can upregulate the expression of IL-1β in pancreatic islets and adipose tissue, resulting in the development of T2DM [104–106]. As a K_ATP_ channel blocker, glibenclamide is the most widely used sulfonylurea drug for the treatment of T2DM, by blocking NLRP3 inflammasome activation [65]. However, to date, the specific mechanisms of NLRP3 inflammasome activation and regulation in T2DM probably have not been fully elucidated.

### Ischemic stroke, T2DM, and the NLRP3 inflammasome

**Fig. 4:**
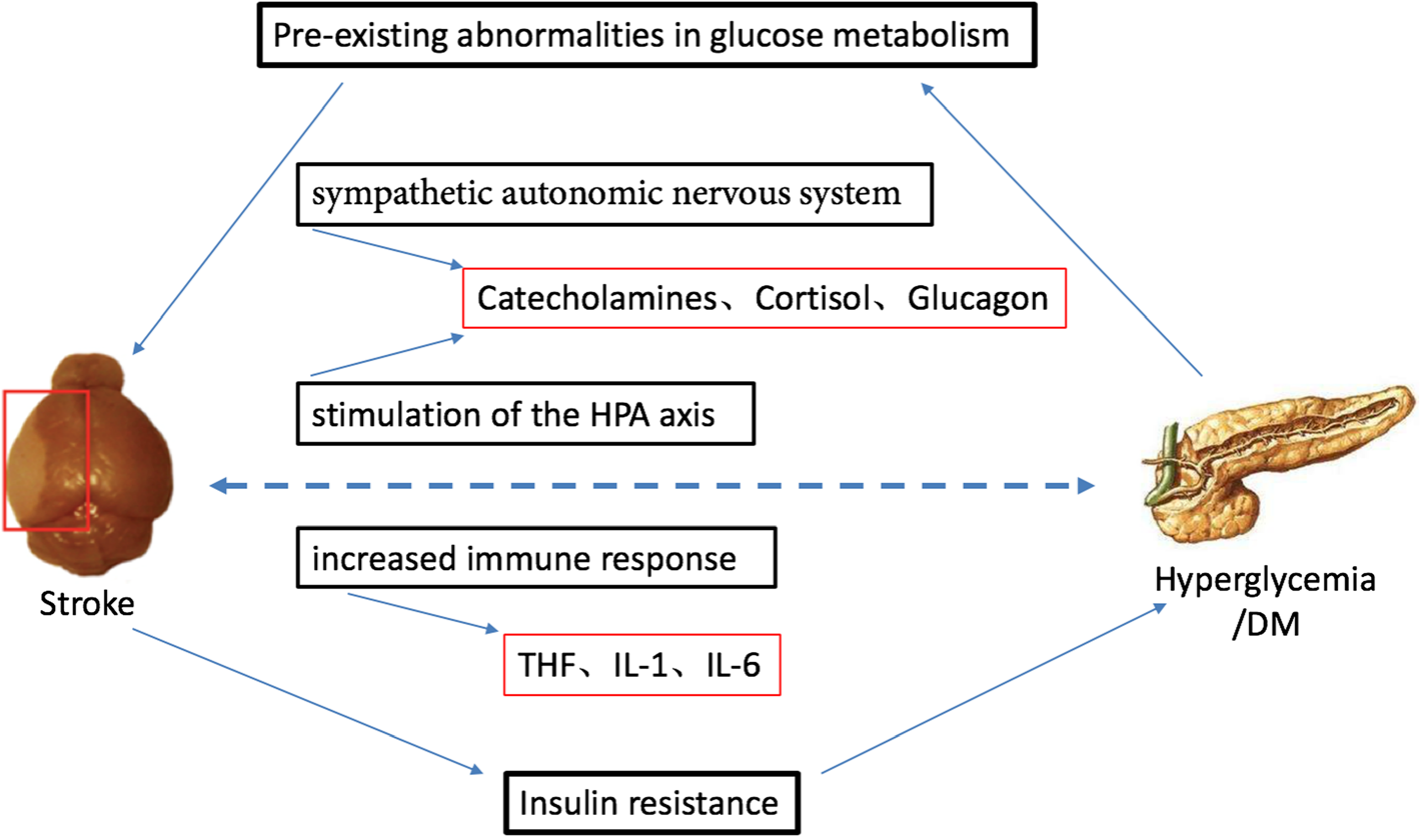
Mechanisms leading to hyperglycemia in ischemic stroke. The high incidence of hyperglycemia after stroke may be due to abnormal glucose metabolism, activation of the hypothalamic pituitary adrenal axis, increased sympathetic nervous system activity, increased stress, and inflammatory response

Diabetic patients with stroke experience significantly greater severity of stroke and worse prognosis. So too, a significant proportion of patients develop elevated blood glucose after acute ischemic stroke. Yong et al. [107] found that blood glucose decreased in the first 24 h after stroke, but increased during the subsequent 24 h. This late stage of hyperglycemia may be associated with impaired glucose metabolism [108]. The cause of hyperglycemia after stroke may be abnormal glucose metabolism, activation of the hypothalamic pituitary adrenal axis leading to increased cortisol levels, and increased sympathetic nervous system activity leading to the release of catecholamines. These factors then promote gluconeogenesis, protein hydrolysis, and lipolysis leading to excessive glucose production [109]. Adrenaline inhibits the binding of insulin to its receptor, and therefore, glucose transport into cells, which leads to insulin resistance [110]. Increased stress and an inflammatory response also significantly aggravate stroke injury in diabetic patients (Fig. 4).

As mentioned above, both diabetes and stroke are closely associated with inflammation, and inflammatory responses have crucial roles in each. There are few studies regarding these diseases and the NLRP3 inflammasome. Our study found that MCC950 can improve prognosis in diabetic mice with stroke [96], which is consistent with other research. MCC950 mitigated Aβ pathology and therefore improved cognition by suppressing NLRP3 inflammasome activation and increasing the phagocytic capability of microglia [111]. Zhai et al. [112] found that inhibition of NLRP3 inflammasome activation may be a potential therapeutic approach for diabetic encephalopathy. MCC950 ameliorated deficits in hippocampal-dependent memory after diabetic-stroke in rats, through inhibition of the NLRP3 inflammasome with decreased IL-1β expression, lower blood-brain barrier permeability, and reduced cell death of the neurons in the CA1 and DG regions of the hippocampus. As a therapy, MCC950 has the potential to prevent neurovascular remodeling and worsened the cognitive decline in diabetic patients after stroke [113]. Based on a comprehensive analysis, it can be concluded that activation of the NLRP3 inflammasome aggravates diabetic stroke. Measures taken against NLRP3 inflammasome activity may be used to treat ischemic stroke that is concomitant with diabetes.

### IL-1β and obesity-induced insulin resistance

Currently, there are ~ 2.3 billion overweight adults in the world, and the World Health Organization alarmingly predicts that the tendency toward obesity will continue to rise [114]. Overnutrition promotes insulin resistance, and being overweight or obese are the primary risk factors of T2DM [14, 115]. The NLRP3 inflammasome has been associated with obesity-induced insulin resistance and pancreas beta cell failure [116]. It is thus logical to suggest that mediators of innate immunity may contribute to the pathological consequences of obesity and diabetes. Obesity can promote the priming signals toward NLRP3 inflammasome formation in diabetes. IL-1β contributes to the pro-inflammatory response in obesity [52, 117].

Stienstra et al. [118] suggested that inflammasome activation is involved from obesity to insulin resistance and finally develop into T2DM.

IL-1β is activated by cleavage of pro-IL-1β, under the stimulation of caspase-1, through the molecular platform of the NLRP3 inflammasome. It has been linked to the etiopathogenesis of several sterile inflammatory diseases such as T2DM, atherosclerosis, and Alzheimer’s disease [119, 120]. IL-1β can come from not only the beta cell itself, but also from blood monocytes that infiltrate the islet. It is the predominant macrophage-derived cytokine, and its levels are elevated in obese patients with T2DM [68, 121, 122]. One study provided evidence of defective production of IL-1β by circulating monocytes that could be due to impaired activation of the NLRP3 inflammasome [20]. In this regard, it may only be effective to target IL-1β in diabetic subjects that involve NLRP3 inflammasome activation. One study by Mirza et al. [123] demonstrated that in diabetic humans and mice, the sustained activity of the NLRP3 inflammasome associated with wounds leads to impaired early healing of these wounds. Recently, Dror et al. [124] showed that postprandial macrophage-derived IL-1β promoted insulin, in patients with T2DM. Using diabetic db/db mice, they found that inhibiting NLRP3 inflammasome activity could improve the healing of wounds.

In the development of obesity and T2DM, NLRs can sense hyperglycemia and then elicit NLRP3 inflammasome-mediated inflammation. Therefore, this shows that hyperglycemia is a strong inducer of NLRP3, and secretion of IL-1β is closely associated with insulin resistance [29, 125, 126]. Loss of weight in obese T2DM individuals is related to the diminution of the NLRP3 inflammasome and IL-1β expression in subcutaneous adipose tissue [127]. By contrast, in diabetes and its complications, while the inflammatory process may not be easily reversible, targeting the NLRP3 inflammasome as an early preventive strategy may prove beneficial. In the development of obesity in mice induced by a high-fat diet, pretreatment with casein hydrolysate showed that NLRP3 inflammasome-mediated IL-1β secretion in adipose tissue could be attenuated [128]. The decline in NLRP3 inflammasome-mediated IL-1β activation also improves obesity-induced insulin resistance. To understand further the effect of the NLRP3 inflammasome in obesity and insulin resistance, Stienstra et al. [129] researched the response of NLRP3^−/−^, ASC^−/−^, and Casp1^−/−^ mice to a high-fat diet. They showed that the NLRP3 inflammasome, in addition to its role in the innate immune response, contributes to obesity-induced insulin resistance.

In addition, altering the cellular metabolic status with statins could promote insulin resistance by activating the NLRP3 inflammasome [130]. Consistent with previous studies, some researchers provided direct in vivo evidence that activation of the NLRP3 inflammasome in diet-induced obesity is essential for causing pancreatic damage. Therefore, to the best of our knowledge, it is an important mechanism of progression toward T2DM [131]. Given that mitochondrial ROS are elevated in obesity, and ROS are implicated in NLRP3 inflammasome assembly, it is possible that mitochondrial dysfunction could influence NLRP3 inflammasome activation that results in pancreatic damage in obese patients [27].

To summarize, these findings indicate that metabolites and stress provide the necessary danger signal for NLRP3 inflammasome activation. However, the exact mechanisms underlying the host’s sensing of obesity and hyperglycemia and how these danger signals trigger the NLRP3 inflammasome remain unclear.

### TXNIP, insulin resistance, and cerebral I/R

Thioredoxin-interacting protein (TXNIP, also called vitamin D3-upregulated protein 1, or VDUP1) is the endogenous inhibitor and regulator of thioredoxin. TXNIP is a signaling molecule that causes the activation of the NLRP3 inflammasome in response to endoplasmic reticulum stress [132]. The TXNIP interaction is a specific feature of NLRP3, binding protein to the NLRP3 inflammasome, and is associated with insulin resistance and multiple organ damage [33]. Simultaneously, hyperglycemia induces high levels of TXNIP [133, 134]. Upregulation of TXNIP induces an increase in oxidative stress that activates the NLRP3 inflammasome. NLRP3 inflammasome signaling is regarded as a potential culprit in obesity-mediated insulin resistance and T2DM. Zhou et al. [33] evaluated the involvement of TXNIP in NLRP3 inflammasome activation. By feeding TXNIP- and NLRP3-deficient mice a high-fat diet, they found that these mice developed impaired glucose homeostasis relative to that of wild-type mice under the same conditions, which was associated with less release of IL-1β from their islet cells. TXNIP deficiency suppressed activation of the NLRP3 inflammasome. This result indicates that TXNIP deficiency protects islet beta cells in vivo.

Akin to the effect of TXNIP on insulin resistance, the role of TXNIP in ischemic stroke showed exacerbated brain injury through redox imbalance and NLRP3 inflammasome activation [135]. Recent evidence has also shown that the therapeutic implication of its inhibition is regulated by nuclear factor erythroid 2-related factor 2 (NRF2), as a key part in the antioxidant stress system, thereby inhibiting NLRP3 inflammasome activation in cerebral I/R [136]. Another study suggested that pretreatment with umbelliferone suppressed the expression of TXNIP and NLRP3 inflammasome, ameliorating cerebral I/R injury. Notably, the same changes in TXNIP and the NLRP3 inflammasome occurred in the rat ischemic brain [80]. This shows that the TXNIP/NLRP3 inflammasome is an important contributor to ischemic stroke. Therefore, this signal pathway may be a novel target for neuroprotection to prevent or treat cerebral ischemic stroke in diabetes.

In general, diabetes exacerbates brain damage after stroke by enhancing the neuroinflammatory signaling cascade, in particular by activation of microglia, leukocytes, adhesion molecules, upregulation of certain pro-inflammatory cytokines, TXNIP, NLRs, and other immune intermediaries.

## Conclusion and future perspective

Both diabetes and ischemia are complex disorders. The disease, comorbidity, and treatments affect long-term outcomes and brain recovery. The NLRP3 inflammasome is well recognized as a key element in T2DM and ischemic stroke. The discovery of the NLRP3 inflammasome has provided a new strategy for investigating the molecular mechanisms of ischemic stroke and T2DM. Inhibiting NLRP3 inflammasome activation may provide insights into future therapies for ischemic stroke accompanied by T2DM.

There remain many questions, especially regarding cross-talk networks between NLRP3 inflammasome activation and the physiological course of diabetes concomitant with ischemic stroke. Although anti-inflammasome drugs have achieved significant effects in animal (mice and pig) experiments [21, 63, 64, 116, 137], clinical evidence is limited [68]. Novel therapies are urgently needed to reduce the risk of ischemic stroke in patients with diabetes. Identification of the mechanisms of activation and regulation of inflammasomes are new targets for controlling inflammation and delaying the progression of diabetes and ischemic stroke.

## Abbreviations

AMPK: Adenosine 5′-monophosphate (AMP)-activated protein kinase
C2TA: Class 2 transcription activator
I/R: Ischemia-reperfusion injury
IL-1β: Interleukin-1β
IRS-1: Insulin receptor substrate-1
MAPK: Mitogen-activated protein kinases
MCAO: Middle cerebral artery occlusion
NAIP: Neuronal apoptosis inhibitor protein
NLR: Nucleotide-binding oligomerization domain-like receptor
NRF2: Nuclear factor erythroid 2-related factor 2
ROS: Reactive oxygen species
STAT1: Signal transducer and activator of transcription 1
T2DM: Type 2 diabetes mellitus
TNF-α: Tumor necrosis-factor-α
TP1: Telomerase-associated protein 1
TXNIP: Thioredoxin-interacting protein
